# Supplementary material for: Angiotensin II Promotes Mitral Valve and Left Atrial Pathology in Fibrillin-1C1041G/+ Mice
Source: bioRxiv. 2026 Oct 8:2026.10.02.755732. Preprint. [Version 1] doi: 10.64898/2026.10.02.755732 (PMC13692065; doi:10.64898/2026.10.02.755732)
Supplement: Supplementary file 1 [file NIHPP2026.10.02.755732V1-supplement-1.pdf]

bioRxiv preprint doi: https://doi.org/10.64898/2026.10.02.755732; this version posted October 8, 2026. The copyright holder for this preprint (which was not certified by peer review) is the author/funder, who has granted bioRxiv a license to display the preprint in perpetuity. It is made available under a CC-BY 4.0 International license.

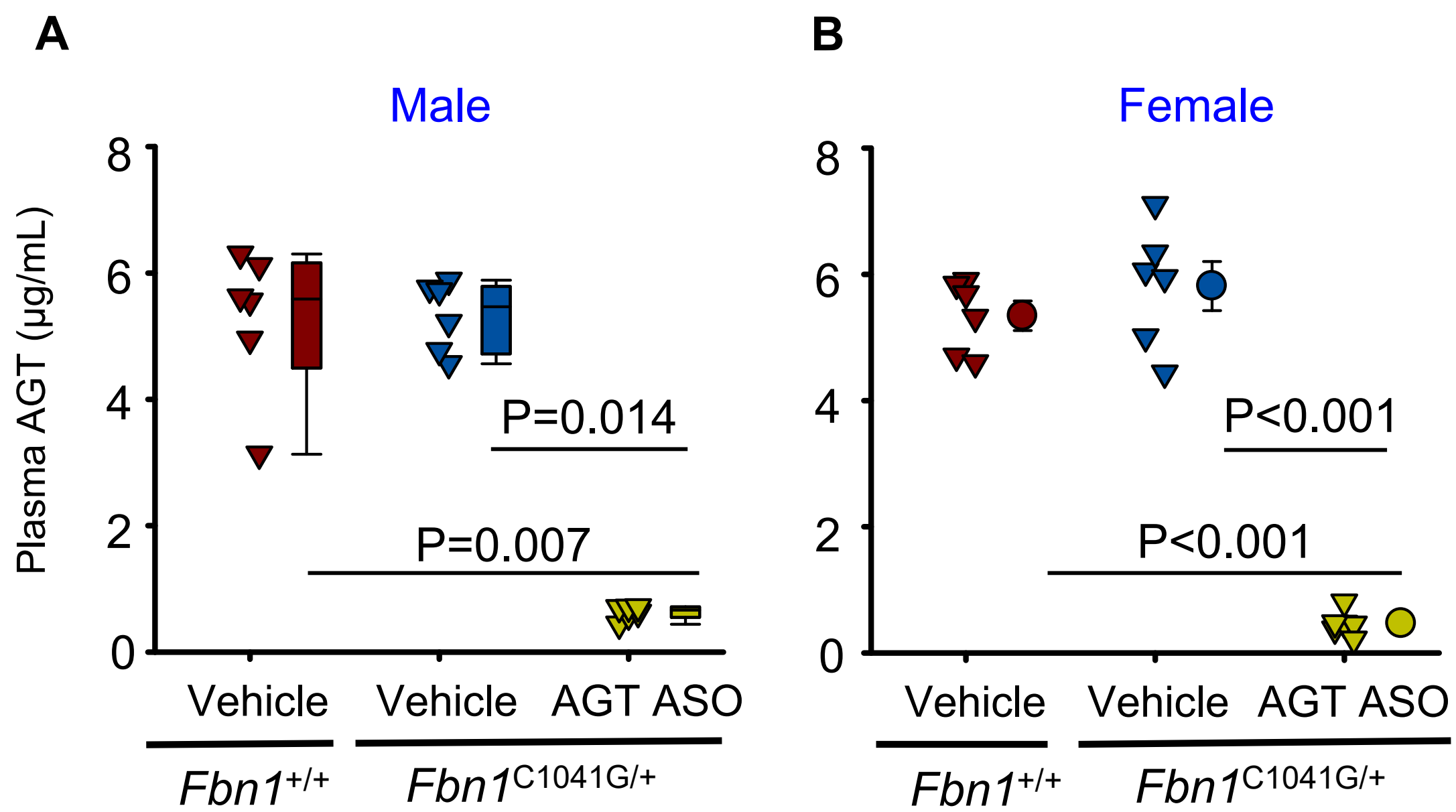

Figure S1. Inhibition of AGT synthesis suppressed plasma AGT concentrations. *Fbn1*$^{+/+}$ mice were administered PBS (vehicle), and *Fbn1*$^{C1041G/+}$ mice were administered vehicle or AGT ASO by subcutaneous injection once weekly for 12 weeks, beginning at 4-5 weeks of age. Plasma AGT concentrations were determined at the endpoint. Data were analyzed using Kruskal-Wallis one-way ANOVA on ranks followed by Dunn's post hoc test for **A** and one-way ANOVA followed by Holm-Sidak post hoc test for **B**. n=6/group.

bioRxiv preprint doi: https://doi.org/10.64898/2026.10.02.755732; this version posted October 8, 2026. The copyright holder for this preprint (which was not certified by peer review) is the author/funder, who has granted bioRxiv a license to display the preprint in perpetuity. It is made available under a CC-BY 4.0 International license.

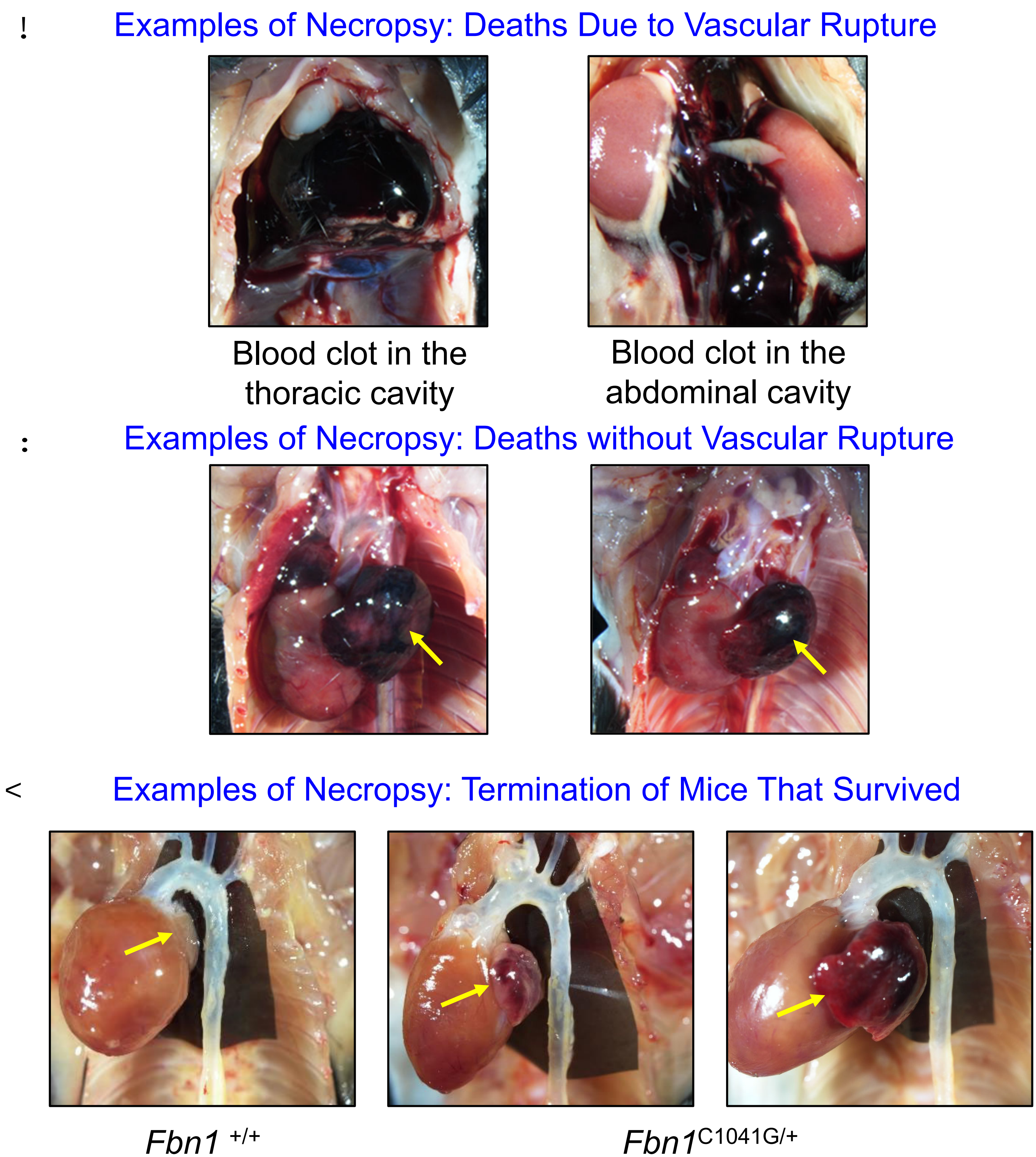

Figure S2. Chronic AngII infusion into *Fbn1*$^{C1041G/+}$ mice provoked left atrial pathology. Male and female *Fbn1*$^{+/+}$ and *Fbn1*$^{C1041G/+}$ mice were infused with AngII (1,000 ng/kg/min) for 4 weeks. In situ images in **A** and **B** are from mice that died before the endpoint. In situ images in **C** show hearts, including the left atrium, from *Fbn1*$^{+/+}$ and *Fbn1*$^{C1041G/+}$ mice. Yellow arrows indicate the left atrium.

bioRxiv preprint doi: https://doi.org/10.64898/2026.10.02.755732; this version posted October 8, 2026. The copyright holder for this preprint (which was not certified by peer review) is the author/funder, who has granted bioRxiv a license to display the preprint in perpetuity. It is made available under a CC-BY 4.0 International license.

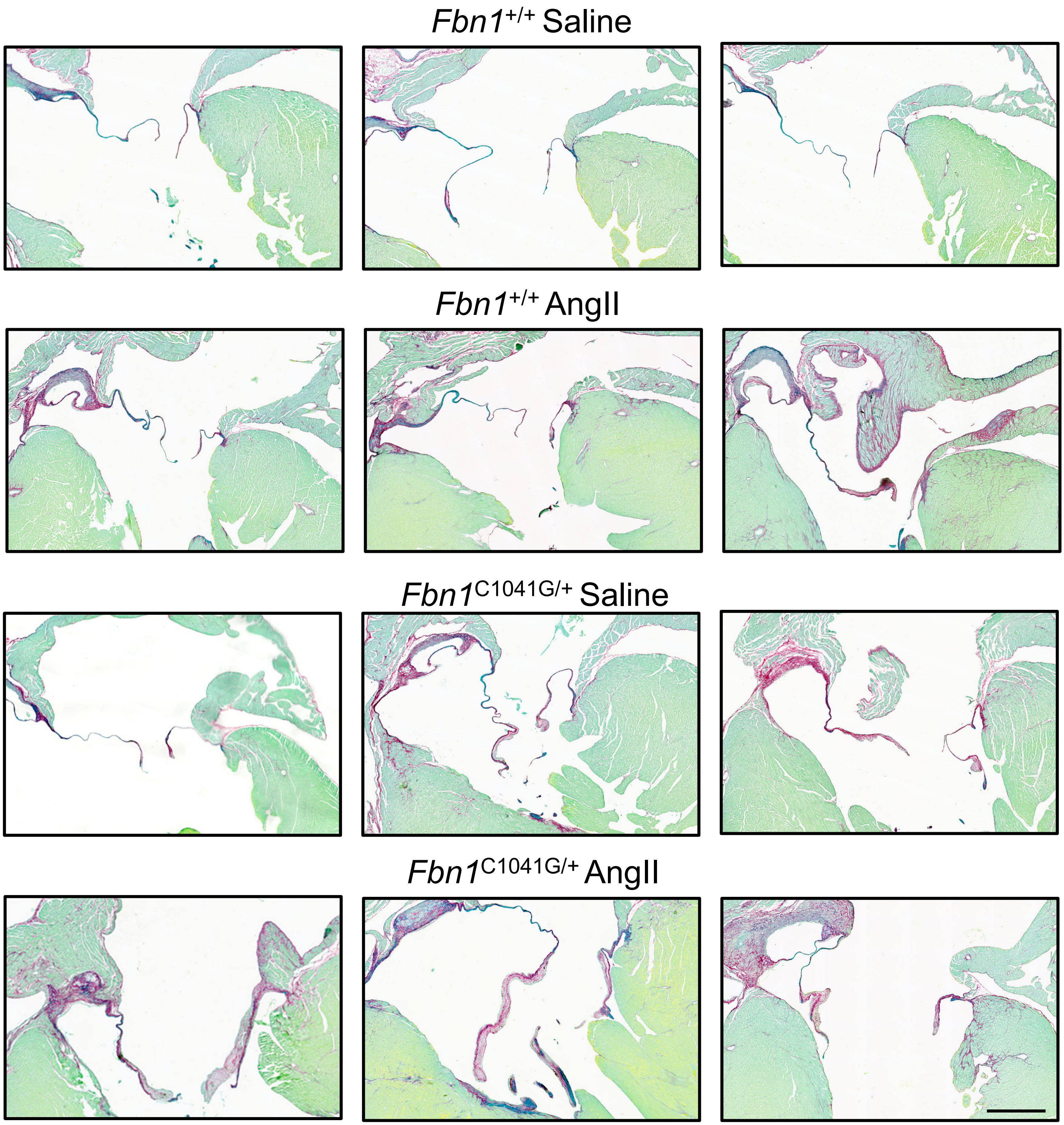

Figure S3. Chronic AngII infusion increased collagen deposition in mitral valves. Examples are shown for *Fbn1*$^{+/+}$ and *Fbn1*$^{C1041G/+}$ mice infused with either saline or AngII (1,000 ng/kg/min) for 4 weeks. Tissue sections were stained with picrosirius red and fast green. Scale bar equals 500 µm.

bioRxiv preprint doi: https://doi.org/10.64898/2026.10.02.755732; this version posted October 8, 2026. The copyright holder for this preprint (which was not certified by peer review) is the author/funder, who has granted bioRxiv a license to display the preprint in perpetuity. It is made available under a CC-BY 4.0 International license.

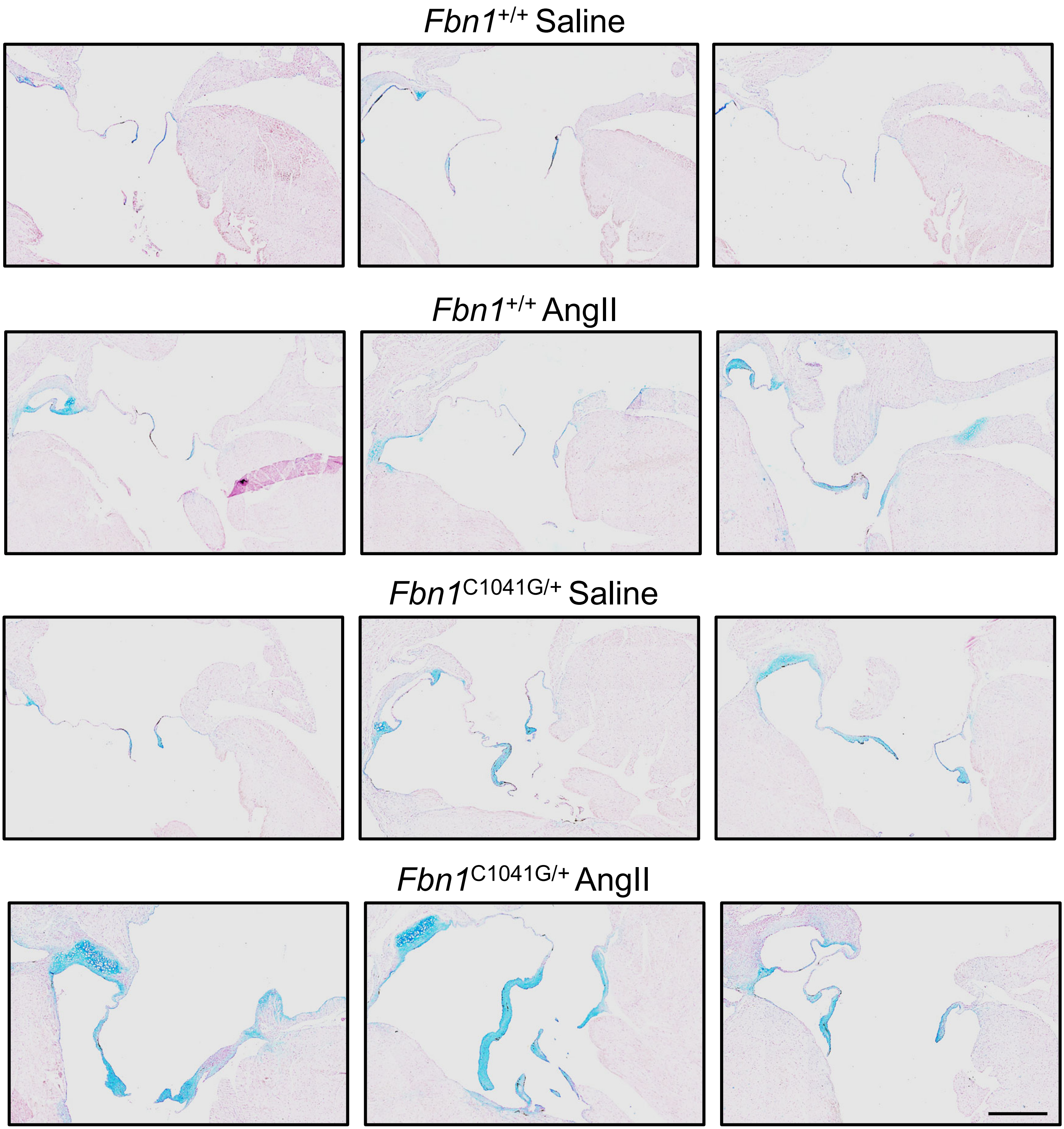

Figure S4. Chronic AngII infusion increased proteoglycan deposition in mitral valves. Examples are shown for *Fbn1*$^{+/+}$ and *Fbn1*$^{C1041G/+}$ mice infused with either saline or AngII (1,000 ng/kg/min) for 4 weeks. Tissue sections were stained with Alcian blue and nuclear red. Scale bar equals 500 μm.

bioRxiv preprint doi: https://doi.org/10.64898/2026.10.02.755732; this version posted October 8, 2026. The copyright holder for this preprint (which was not certified by peer review) is the author/funder, who has granted bioRxiv a license to display the preprint in perpetuity. It is made available under a CC-BY 4.0 International license.

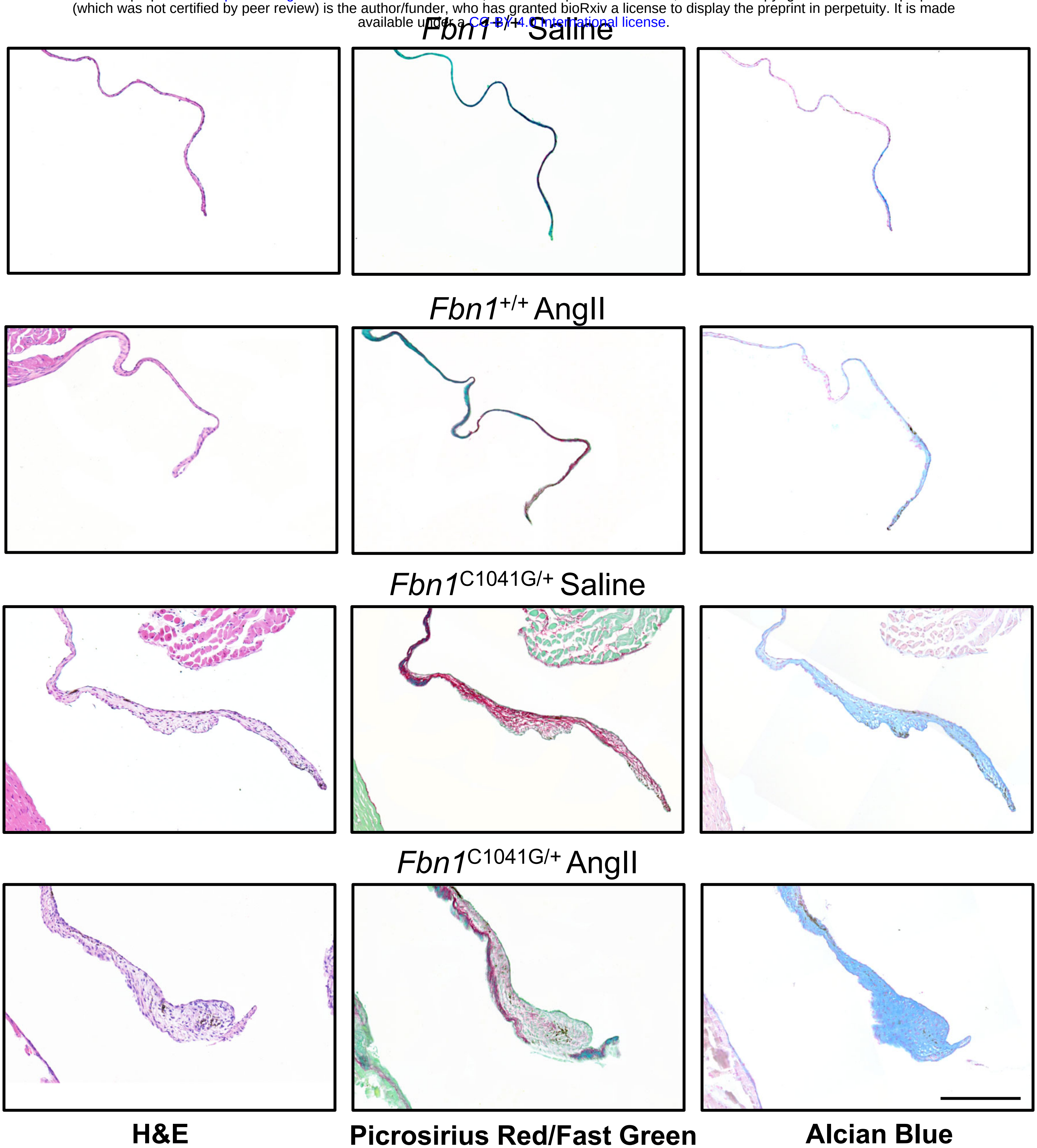

Figure S5. Images of anterior mitral valve leaflet. Examples are shown for *Fbn1*$^{+/+}$ and *Fbn1*$^{C1041G/+}$ mice infused with either saline or AngII (1,000 ng/kg/min) for 4 weeks. Tissue sections were stained with hematoxylin/eosin, picrosirius red/fast green, or Alcian blue/nuclear red. Scale bar equals 200 μm.

bioRxiv preprint doi: https://doi.org/10.64898/2026.10.02.755732; this version posted October 8, 2026. The copyright holder for this preprint (which was not certified by peer review) is the author/funder, who has granted bioRxiv a license to display the preprint in perpetuity. It is made available under a CC-BY 4.0 International license.

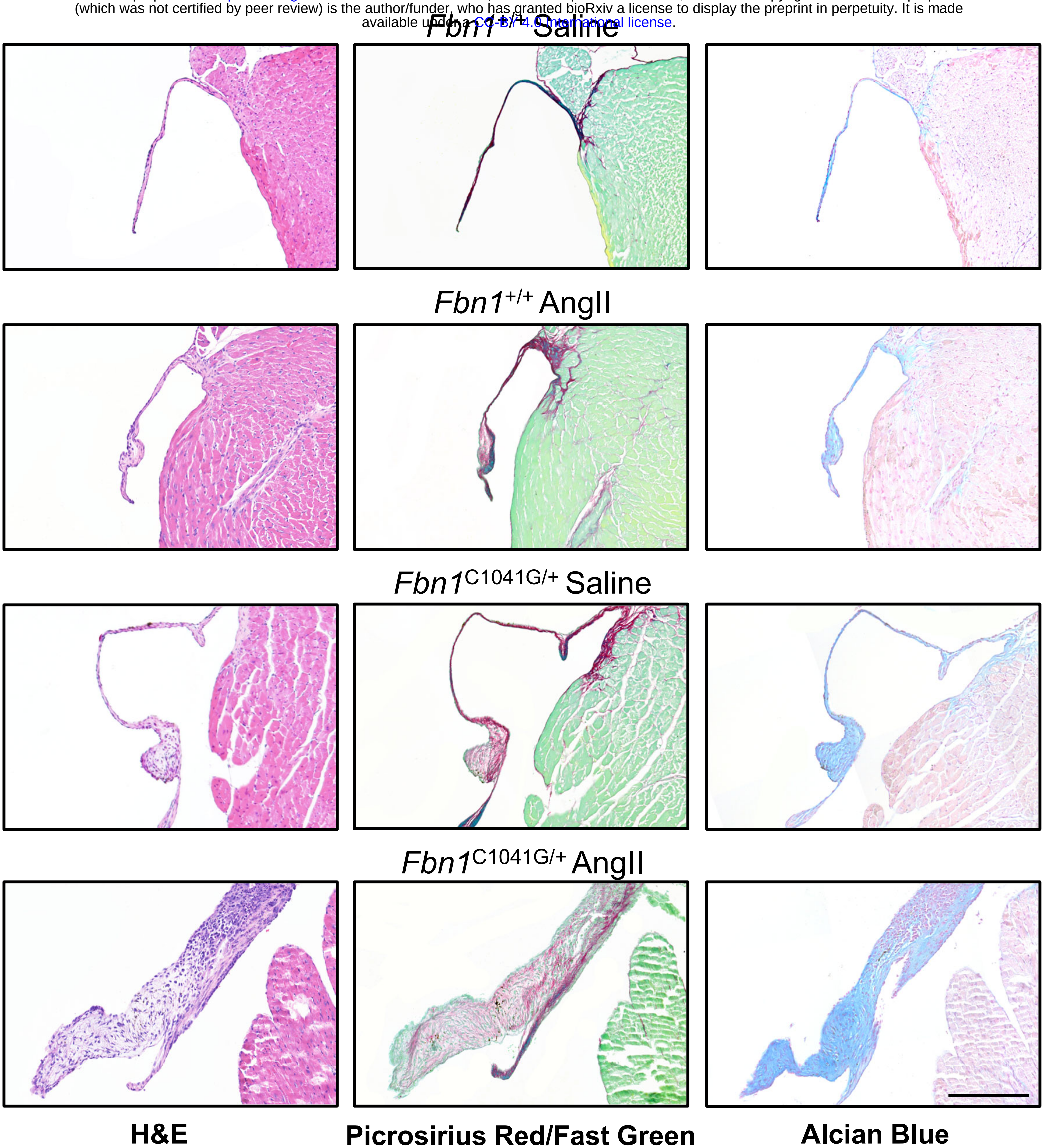

Figure S6. Images of posterior mitral valve leaflet. Examples are from *Fbn1*$^{+/+}$ and *Fbn1*$^{C1041G/+}$ mice infused with either saline or AngII (1,000 ng/kg/min) for 4 weeks. Tissue sections were stained with hematoxylin/eosin, picrosirius red/fast green, or Alcian blue/nuclear red. Scale bar equals 200 μm.

bioRxiv preprint doi: https://doi.org/10.64898/2026.10.02.755732; this version posted October 8, 2026. The copyright holder for this preprint (which was not certified by peer review) is the author/funder, who has granted bioRxiv a license to display the preprint in perpetuity. It is made available under a CC-BY 4.0 International license.

Angiotensin II Promotes Mitral Valve and Left Atrial Pathology in Fibrillin-1$^{C1041G/+}$ Mice

Michael K. Franklin,[1] Deborah A. Howatt,[1] Alex C. Pettey,[1,2]
Jessica J. Moorleghen,[1] Bryana M. Levitan,[3] Adam E. Mullick,[4]
Jeffrey E. Saffitz,[5] Hong S. Lu,[1-3] Alan Daugherty[1-3]

[1] Saha Cardiovascular Research Center
[2] Saha Aortic Center
[3] Department of Physiology
University of Kentucky, Lexington, KY

[4] Ionis Pharmaceuticals, Inc, Carlsbad, CA

[5] Department of Pathology, Beth Israel Deaconess Medical Center, Harvard Medical School, Boston, MA

Running Title: FBN1, Mitral Valve, and Atrial Pathology

Corresponding Authors:
Alan Daugherty
Saha CVRC, BBSRB, Room 243
University of Kentucky
741 S Limestone
Lexington, KY 40536-0509
e-mail: Alan.Daugherty@uky.edu

Hong S. Lu
Saha CVRC, BBSRB, Room 249
University of Kentucky
741 S Limestone
Lexington, KY 40356-0509
e-mail: Hong.Lu@uky.edu

bioRxiv preprint doi: https://doi.org/10.64898/2026.10.02.755732; this version posted October 8, 2026. The copyright holder for this preprint (which was not certified by peer review) is the author/funder, who has granted bioRxiv a license to display the preprint in perpetuity. It is made available under a CC-BY 4.0 International license.

Table S1. Necropsy on *Fbn1*$^{C1041G/+}$ mice represented in Figure 1C-F that died during AngII infusion.

**Sex**	**From Initiation of AngII Infusion to Death**	**Cause of Death (Number of Mice)**		
	Time (Days)	Blood Clot Found in Thoracic Cavity	Blood Clot Found in Abdominal Cavity	Nonvascular-based Death
**Male**	1		X	
	1		X	
	2		X	
	3		X	
	5		X	
	14			X
	21			X
	**Total**	**0**	**5**	**2**
**Female**	5	X		
	6	X		
	9			X
	**Total**	**2**	**0**	**1**

bioRxiv preprint doi: https://doi.org/10.64898/2026.10.02.755732; this version posted October 8, 2026. The copyright holder for this preprint (which was not certified by peer review) is the author/funder, who has granted bioRxiv a license to display the preprint in perpetuity. It is made available under a CC-BY 4.0 International license.

Table S2. Echocardiography in mice infused with angiotensin II (AngII)

Males	Fbn1$^{+/+}$ (mean ± SEM)	Fbn1$^{C1041G/+}$ (mean ± SEM)	P value	Statistical Test
HR (bpm)	519 ± 23	503 ± 18	-	-
LVID;d (mm)	4.2 ± 0.3	4.6 ± 0.5	0.053	Student's *t* test
LV mass (mg)	90 ± 7	118 ± 8	0.016	Student's *t* test
LVAW;d (mm)	0.68 ± 0.05	0.77 ± 0.04	0.20	Student's *t* test
LVPW;d (mm)	0.57 ± 0.03	0.62 ± 0.04	0.37	Student's *t* test
EF (%)	49 ± 3	47 ± 2	0.74	Student's *t* test
CO (mL/min)	20 ± 2	23 ± 2	0.16	Student's *t* test

Females	Fbn1$^{+/+}$ (mean ± SEM) OR (median, 25th %, 75th %)	Fbn1$^{C1041G/+}$ (mean ± SEM) OR (median, 25th %, 75th %)	P value	Statistical Test
HR (bpm)	463 ± 28	504 ± 18	-	-
LVID;d (mm)	3.8 ± 0.1	4.0 ± 0.3	0.52	Student's *t* test
LV mass (mg)	99 ± 4	128 ± 13	0.061	Welch's *t* test
LVAW;d (mm)	0.83 ± 0.04	0.99 ± 0.07	0.059	Student's *t* test
LVPW;d (mm)	0.67 ± 0.05	0.71 ± 0.03	0.52	Welch's *t* test
EF (%)	50 ± 3	52 ± 4	0.68	Student's *t* test
CO (mL/min)	13, 11, 17	16, 15, 19	0.10	Rank sum test

Male and female *Fbn1*$^{+/+}$ and *Fbn1*$^{C1041G/+}$ mice, 8–10 weeks of age, were infused with AngII (1,000 ng/kg/min) for 4 weeks. Cardiac structure and function were assessed by echocardiography before study termination.

Abbreviations

Abbreviation	Full Name
HR	Heart Rate
LVID;d	Left Ventricle Internal Diameter; Diastole
LV mass	Left Ventricular Mass
LVAW;d	Left Ventricle Anterior Wall; Diastole
LVPW;d	Left Ventricle Posterior Wall; Diastole
EF	Ejection Fraction
CO	Cardiac Output

bioRxiv preprint doi: https://doi.org/10.64898/2026.10.02.755732; this version posted October 8, 2026. The copyright holder for this preprint (which was not certified by peer review) is the author/funder, who has granted bioRxiv a license to display the preprint in perpetuity. It is made available under a CC-BY 4.0 International license.

MAJOR RESOURCES TABLES

Table I. Mouse Models Used in This Study

Strain	Vendor	Background Strain	Sex	Other Information	Persistent ID/URL
Fbn1$^{C1041G/+}$	JAX (Strain # 012885)	C57BL/6J	Male and Female	Breeding Scheme:<br>Male *Fbn1*$^{C1041G/+}$ x Female *Fbn1*$^{+/+}$<br>Offspring:<br>*Fbn1*$^{+/+}$ and *Fbn1*$^{C1041G/+}$ littermates	https://www.jax.org/strain/012885
Fbn1$^{+/+}$					

Table II. Reagents for Histology

Reagent	Vendor	Cat #	Working Concentration
Antigen retrieval solution	Biogenex	HK547-XAK	Ready-to-use
Eosin Y	abcam	AB246824	Ready-to-use
Goat serum	Vector Laboratories	MP-7451	2.5% vol/vol
Hydrogen peroxide	Fisher Scientific	H325-500	1% vol/vol
Limonene	Medical Chemical Corporation	6533A	Ready-to-use
Mayer's hematoxylin	Electron Microscopy Sciences	26043-06	Ready-to-use
NovaRED substrate kit	Vector Laboratories	SK-4805	Ready-to-use

Table III. Primary Antibodies for Immunostaining

Antibody	Vendor	Cat #	Working Concentration
Rabbit anti-CD68 (E3O7V)	Cell Signaling Technology	97778	0.1 µg/mL
Rabbit anti-CD41 antibody	Proteintech	24552-1-AP	0.3 µg/mL

Table IV. Secondary Antibodies for Immunostaining

Antibody	Vendor	Cat #	Note
ImmPress Goat Anti-Rabbit IgG	Vector	MP-7451	No concentration information available; Ready-to-use (per manufacturer)

Table V. Animal Study Information Following the ARRIVE Essential 10

Figure 1A and S1A

Groups	Sex	Age (weeks)*	Number (Start)	Number (Termination)
Fbn1$^{+/+}$ PBS	Male	4-5	6	6
Fbn1$^{C1041G/+}$ PBS	Male	4-5	6	6
Fbn1$^{C1041G/+}$ AGT ASO	Male	4-5	6	6

Figure 1B and S1B

Groups	Sex	Age (weeks)*	Number (Start)	Number (Termination)
Fbn1$^{+/+}$ PBS	Female	4-5	6	6
Fbn1$^{C1041G/+}$ PBS	Female	4-5	6	6
Fbn1$^{C1041G/+}$ AGT ASO	Female	4-5	6	6

Figure 1C and D

Groups	Sex	Age (weeks)*	Number (Start)	Number (Termination)
Fbn1$^{+/+}$ Saline	Male	8-10	5	5
Fbn1$^{+/+}$ AngII	Male	8-10	5	5
Fbn1$^{C1041G/+}$ Saline	Male	8-10	6	6
Fbn1$^{C1041G/+}$ AngII	Male	8-10	12	5

bioRxiv preprint doi: https://doi.org/10.64898/2026.10.02.755732; this version posted October 8, 2026. The copyright holder for this preprint (which was not certified by peer review) is the author/funder, who has granted bioRxiv a license to display the preprint in perpetuity. It is made available under a CC-BY 4.0 International license.

Figure 1E and F

Groups	Sex	Age (weeks)*	Number (Start)	Number (Termination)
Fbn1$^{+/+}$ Saline	Female	8-10	6	6
Fbn1$^{+/+}$ AngII	Female	8-10	5	5
Fbn1$^{C1041G/+}$ Saline	Female	8-10	7	7
Fbn1$^{C1041G/+}$ AngII	Female	8-10	10	7

Figure 1G

Groups	Sex	Age (weeks)*	Number (Start)	Number (Termination)
Fbn1$^{+/+}$ Saline	Male	8-10	5	5
Fbn1$^{+/+}$ AngII	Male	8-10	5	5
Fbn1$^{C1041G/+}$ Saline	Male	8-10	5	5
Fbn1$^{C1041G/+}$ AngII	Male	8-10	5	5

Figure 1H

Groups	Sex	Age (weeks)*	Number (Start)	Number (Termination)
Fbn1$^{+/+}$ Saline	Female	8-10	4	4
Fbn1$^{+/+}$ AngII	Female	8-10	6	6
Fbn1$^{C1041G/+}$ Saline	Female	8-10	4	4
Fbn1$^{C1041G/+}$ AngII	Female	8-10	7	7

ARRIVE Essential 10 Checklist

Item	Application
Ethics	Approved by the University of Kentucky IACUC (Protocol #: 2018-2967).
Sex	Described in each figure.
Inclusion criteria	Based on sex, age, body weight, and overall health appearance in each experiment.
Exclusion criteria	Based on inconsistent genotypes between pre-study and post-study verification, and medical cases reported by a veterinarian.
Sample size	Described in Major Resources Tables
Sample size calculation	None
Primary endpoint	Mitral valve thickness
Randomization	Study mice were numbered and grouped randomly based on their genotype and sex.
Blinding	Maximal mitral valve thickness and ultrasound parameters were measured by an investigator blinded to the study group assignment. The data were independently verified by a staff member.
Statistical analysis	SigmaPlot 16.0 (SYSTAT Software Inc., CA)
Statistical method	Described in each figure legend.
Data availability	All numerical data used for figures and supplemental figures are available in the Supplemental Excel File.
